# Unusual location of gouty arthritis with shoulder joint involvement in an elderly male patient: a rare case report

**DOI:** 10.1097/MS9.0000000000000389

**Published:** 2023-03-27

**Authors:** Oadi N. Shrateh, Afnan W.M. Jobran, Momen A. Zaid, Heba K. Hamed, Mohammad Y. Asees, Omar Soboh, Ahmad A. Dallashi

**Affiliations:** a Medical Research Club, Faculty of Medicine; b Faculty of Medicine, Al-Quds University, Jerusalem; c Department of Internal Medicine, Palestinian Medical Complex; d Professional Medical Laboratories, Department of Clinical chemistry and Hematology, Ramallah, Palestine

**Keywords:** case report, gouty arthritis, shoulder

## Abstract

Gout is a metabolic disease characterized by recurrent episodes of acute arthritis. Gout has been reported in many locations but is rarely localized in the shoulder joint.

**Case presentation:**

A 73-year-old man who visited an outpatient clinic with the main complaint of a right shoulder ache lasting 2 weeks came to our attention. The patient reports his discomfort as being of an unbearable character, happening largely at night and preventing him from falling asleep. In the previous 6 months, he had two episodes of the same ailment that lasted around 3–5 days each and spontaneously resolved. Due to the pain’s continuance without improvement, the patient now seeks medical assistance. Gout with right shoulder involvement was identified as the cause. Prednisolone 40 mg/day for 10 days, allopurinol 300 mg/day, and colchicine 0.5 mg/day were all prescribed for the patient. After 6 months of follow-up, the patient had significantly improved.

**Discussion and conclusions:**

The condition of gout affecting the shoulder joint is quite rare. According to past medical history and clinical manifestations, doctors and orthopedic surgeons should take gouty shoulder arthritis into consideration when there is serious erosion.

## Introduction

Highlights

- The formation of monosodium urate crystals in the synovial fluid of joints and soft tissue is a hallmark of gout.
- Gout typically affects the elbows, knees, feet, and hands; it is relatively uncommon to develop gout in the shoulder joint.
- Gouty arthritis should be taken into account in clinical work when patients with shoulder discomfort, weakness, and limited mobility present, especially when such individuals lack the outward signs of acute arthritis.

The formation of monosodium urate crystals in the synovial fluid of joints and soft tissue is a hallmark of gout. Nearly 2% of people in industrialized nations are affected by it, and it is significantly more prevalent in people who have had kidney transplants^1^,^2^ since it is linked to both lower patient and death-censored graft survival^3^. Although the axial spine has been reported to be affected, the peripheral joints are typically the focus of gout^4^. Although it is unclear how common axial gout is, computed tomography-graphic evidence was found in 35% of individuals who had a history of chronic, poorly managed gout^5^. The primary risk factor for repeated gout attacks is hyperuricemia, which is more common in mature men and is connected to the use of diuretics and alcohol, particularly beer and spirits^6^. Serum uric acid levels exceeding 6.8 mg/dl may cause supersaturation and the crystallization of monosodium urate, which, when deposited in joints and soft tissue, results in gout attacks^3^,^4^. Due to kidney disease and exposure to cyclosporine, both of which raise serum uric acid levels, transplant recipients are considerably more at risk^3^. Monosodium urate crystals cause an inflammatory response, resulting in swollen, tender, and hot joints. Shoulder gout manifests as tophaceous deposits in the rotator cuff, intraosseous tophi in the humeral head, and tophi in the bursa surrounding the shoulder joint, according to the literature^7^. Gout may be misdiagnosed as degenerative, infectious, or malignant arthritis during the clinical diagnosis and treatment process, leading to delays in diagnosis and treatment. Only four cases of shoulder gout have been reported in the English-language literature to date, according to PubMed, and we provide a brief literature review on shoulder gout^7^–^10^. This case report has been reported in line with the SCARE (Surgical CAse REport) Criteria^11^.

## Case presentation

**Figure 1:**
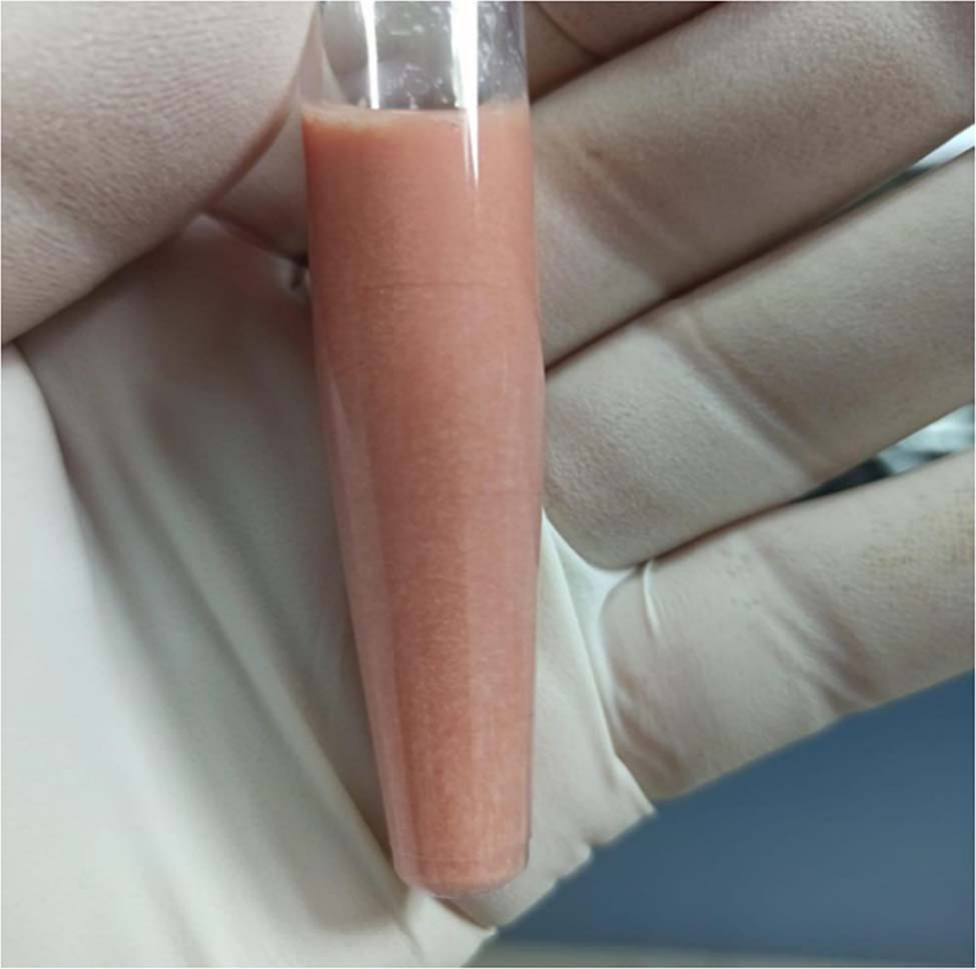
Physical examination of the aspirated synovial fluid of the right shoulder shows a pinkish color with low viscosity and high turbidity.

**Figure 2:**
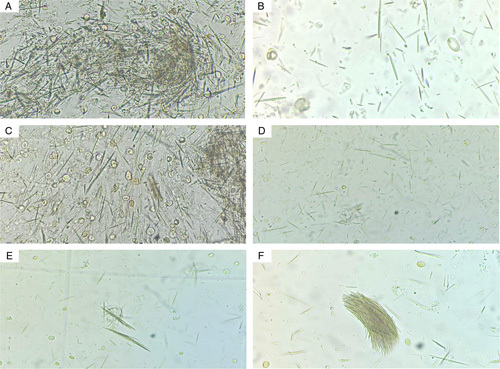
A-F, Microscopic examination of the aspirated synovial fluid of the right shoulder shows monosodium urate crystal with a needle shape. The analysis of fluid shows markedly increased monosodium urate crystal. The uric acid in the fluid is 15 mg/dl with normal sugar and protein content. The white blood cells count is 2500 cell/μl and the reb blood cells count is 26 000 cell/μl.

Our patient is a nonsmoker, 73-year-old male who came to our attention after presenting to a medical outpatient clinic with the chief complaint of right shoulder pain with a 2-week duration. The patient describes his pain as excruciating in nature, mostly occurring at night and awakening him from sleep. He experienced two episodes of the same complaint lasting about 3–5 days in the last 6 months, both with spontaneous resolution. Nowadays, the patient is seeking medical attention due to the persistence of the pain without any improvement. He mentioned that he is consuming large and frequent meals of red meats and some kinds of seafood such as tuna. Past medical history is positive for controlled hypertension, diabetes mellitus, and mild chronic kidney disease. The patient reported no personal and/or family history of cancer, any acute, repeat, or discontinued medications, any allergies, autoinflammatory diseases, any genetic or psychosocial issues, and a significant past surgical history for coronary artery bypass graft. He denies any history of trauma in general or specifically to the shoulder. Upon admission, the physical assessment was unremarkable except for right shoulder pain and tenderness with movement in the absence of redness, swelling, and hotness. Laboratory evaluation revealed C-reactive protein of 111 mg/l (normal range 0–10 mg/l), erythrocyte sedimentation rate of 122 mm/h (normal range 0–15 mm/h), blood urea nitrogen of 32 mg/dl (normal range 8–23 mg/dl), creatinine of 1.4 mg/dl (normal range 0.7–1.2 mg/dl), uric acid of 15 mg/dl (normal range 3.7–7.1 mg/dl), and a normal white blood cell count. Radiological imaging of the right shoulder was normal. An arthrocentesis was performed, and analysis of the aspirated synovial fluid revealed the presence of yellow needle-shaped monosodium urate crystals under a light microscope (Figs. 1, 2) along with a white blood cell count of 2500 cell/μl. A diagnosis of gout with right shoulder involvement was established. The patient was prescribed prednisolone 40 mg/day for 10 days, allopurinol 300 mg/day, and colchicine 0.5 mg/day. The patient was followed up for 6 months with significant improvement. The patient also had a good tolerance for pharmacological agents without any reported complications or adverse events.

## Discussion

Monosodium urate crystal formation in joints and connective tissue is a characteristic of the widespread inflammatory joint disease known as gout. Gout typically affects the elbows, knees, feet, and hands; it is relatively uncommon to develop gout in the shoulder joint^12^. We described a unique incidence of right shoulder joint gouty arthritis.

Acute gouty arthritis, the intercritical phase, and chronic tophaceous gout are the four clinical stages of gout, respectively^13^. Acute gouty arthritis is typically characterized by abrupt, severe pain, swelling, redness, and tenderness in the joints. Poorly managed gout may progress to chronic tophaceous gout, and persistent gout may exhibit more unusual symptoms. A serious joint injury can result from the prolonged deposition of monosodium urate crystals in joints and other bodily tissues, which can present in a variety of ways. These unusual presentations are probably the result of the complexity of the causes^13^.

The preservation of joint space, dense nodules of soft tissues that are occasionally calcified, and punched-out erosions with overhanging cortex and sclerotic edges are radiological features of chronic gouty arthritis^14^. According to the calcium concentration within a tophus, the typical MRI symptoms of tophaceous gouty arthritis include homogenous intermediate signal intensity on T1-weighted images and heterogeneous intermediate-to-low signal intensity on T2-weighted images^15^. In addition to rare instances of coexisting gout and septic shoulder arthritis, there are several examples of gouty shoulder arthritis in the literature. The first example of subacromial impingement brought on by tophaceous rotator cuff gout was described by O’leary *et al*. On the MRI, there was nothing abnormal but supraspinatus tendonitis. The supraspinatus and subscapularis tendons had tophaceous deposits, according to arthroscopic results^7^. A case of tophaceous gout involving the rotator cuff was described by Chang *et al*. Following a shoulder injury, the patient in this instance complained of sporadic pain and a constrained range of motion in the right shoulder. The right shoulder plain radiograph revealed a little amorphous opacity above the humeral head. Urate crystal deposits were discovered by MRI in intrasubstance regions and on the supraspinatus tendon’s articular surface^8^.

After arthroscopic rotator cuff surgery, Ichiseki *et al*. observed the coexistence of gouty and septic shoulder arthritis. Following surgery, the patient experienced a high temperature along with left shoulder pain, edema, and warmth. The study did not, however, discuss the shoulder joint’s imaging manifestations^9^. Right shoulder joint tophaceous gout was described by Tierra Rodriguez *et al*. When performing particular motions and laying on his affected shoulder at night, the patient complained of a right shoulder ache. A plain radiograph of the shoulder joint revealed an irregular opacity occupying the subacromiodeltoid bursa and a punched-out eccentric bony erosion in the clavicle region of the right acromioclavicular joint. Tophi deposits were visible on MRI scans in the subacromiodeltoid bursa and along the upper ridge of the distal end of the clavicle^10^.

Gouty arthritis should be taken into account in clinical work when patients with shoulder discomfort, weakness, and limited mobility present, especially when such individuals lack the outward signs of acute arthritis and have substantial bone loss in the shoulder joint^16^. It can be challenging to distinguish gouty arthritis from osteomyelitis or infectious arthritis. Other potential causes, such as infection, neoplasia, and a condition that destroys bones, must be ruled out^16^. The following ailments need to be taken into account during differential diagnosis: Septic shoulder arthritis is characterized by acute inflammation, redness, swelling, and discomfort. Laboratory testing shows elevated levels of neutrophils and white blood cells, and joint fluid analysis will reveal pus cells or microorganisms^16^. Pain, dysfunction, muscle atrophy, and fistula are the signs of tuberculous shoulder arthritis. Patients also exhibit systemic symptoms of tuberculosis and are Xpert-test positive^17^.

## Conclusion

Rarely does gout affect the shoulder joint, and the bone tissue of the shoulder joint rarely experiences osteolytic damage. It is simple to misdiagnose gouty arthritis of the shoulder with extensive bone degeneration as a shoulder tumor or infectious disease. Conclusion: Despite the rarity of gouty shoulder arthritis, doctors and orthopedic surgeons should take the possibility that gout may cause severe lesions that resemble an infection or neoplastic disease into consideration when treating patients who have a history of the condition, atypical manifestations, or glenoid erosive lesions. This case study sought to inform medical professionals of the unique gouty arthritis symptoms and presentations that could go unnoticed if there is no suspicion.

## Ethical approval

Our institution has exempted this study from ethical review.

## Patient consent

Written informed consent was obtained from the patient for publication of this case report. A copy of the informed consent is available for review by the Editor-in-Chief of this journal on request.

## Sources of funding

This research did not receive any specific grant from funding agencies in the public, commercial, or not-for-profit sectors.

## Author contribution

O.N.S., A.W.M.J., and M.A.Z.: writing the manuscript; M.Y.A. and O.S.: imaging description; H.K.H. and A.A.D.: reviewing and editing the manuscript.

## Conflicts of interest disclosure

The authors declare that they have no known competing financial interests or personal relationships that could have appeared to influence the work reported in this paper.

## Research registration unique identifying number (UIN)

None.

## Guarantor

Oadi N. Shrateh.

## Provenance and peer review

Not commissioned, externally peer-reviewed.

## Authorship

All authors attest that they meet the current ICMJE criteria for authorship.
